# Partial Preservation of Segment IV Confers No Benefit When Performing Extended Right Hepatectomy for Colorectal Liver Metastases

**DOI:** 10.1155/2013/458641

**Published:** 2013-12-11

**Authors:** M. G. Wiggans, S. Fisher, H. Adwan, S. Aroori, M. J. Bowles, D. A. Stell

**Affiliations:** ^1^Hepatopancreatobiliary Surgery, Plymouth Hospitals NHS Trust, Derriford Hospital, Derriford Road, Plymouth, Devon PL6 8DH, UK; ^2^Peninsula College of Medicine and Dentistry, University of Exeter and Plymouth University, John Bull Building, Plymouth, Devon PL6 8BU, UK

## Abstract

*Introduction*. Reducing the volume of resected liver parenchyma may lead to lower morbidity and mortality. The aim of this study was to determine whether partial preservation of segment IV leads to improved outcomes when undertaking extended right hepatectomy for colorectal liver metastases (CRLM). *Materials and Methods*. A retrospective analysis of patients undergoing right-sided hepatectomy for CRLM was performed. Rates of 90-day mortality and organ dysfunction were compared in 117 patients undergoing right hepatectomy (*n* = 85), partially extended right hepatectomy with preservation of part of segment IV (*n* = 20), and fully extended right hepatectomy (*n* = 12). *Results*. The 90-day mortality rate of those undergoing right hepatectomy (3/85) was similar to that of those undergoing extended right hepatectomy (0/12) (*P* = 1.000) but lower than that of those undergoing partially extended right hepatectomy (4/20) (*P* = 0.024). The rates of hepatic and renal dysfunction were similar between patients undergoing right hepatectomy, partially extended or extended hepatectomy. *Discussion*. Preservation of part of segment IV confers little clinical benefit when performing extended right hepatectomy for CRLM.

## 1. Introduction

Extended right hepatectomy is indicated for solitary tumours involving the midplane of the liver and for multifocal tumours involving both the right lobe of the liver and segment IV. The Brisbane classification defines an extended right hepatectomy as the removal of the right lobe of the liver plus all of segment IV up to the umbilical fissure [1]. This procedure removes the most functioning liver (5 or 6 segments) of all liver resections and exposes patients to a significant risk of posthepatectomy liver failure (PHLF), which is associated with a high mortality, especially when complicated by renal dysfunction [2]. The reported incidence of PHLF after extended right hepatectomy is between 1.2% and 32% in published series [3–10]. Lactic acid produced during anaerobic metabolism is cleared by the liver [11] and hyperlactataemia has been shown to predict postoperative liver dysfunction [12].

As the blood supply and biliary drainage of segment IV is provided via the umbilical fissure [13] it is often technically possible to preserve part of segment IV with an intact blood supply and biliary drainage when performing extended right hepatectomy. Although segment IV accounts for only 20% of total liver volume [14] this proportion more than doubles in a right hepatectomy when only segments I–IV remain. Therefore, preservation of part of segment IV may potentially increase the volume of functioning liver parenchyma and reduce the risk of PHLF when undertaking an extended right hepatectomy. Parenchymal-sparing liver resection has been shown to be of benefit in the context of surgery for colorectal metastases [15, 16]. However, the venous drainage of segment IV is provided mainly by the middle hepatic vein, which lies along the midplane of the liver and may be excised during a partially extended right hepatectomy, although some additional venous drainage is provided by smaller veins [17]. The residual part of segment IV may be perfused and have adequate biliary drainage but the venous drainage may be insufficient leading to engorgement and poor function. In addition, due to the pyramidal shape of segment IV [13] subsegment IVA is small and may add little extrafunctional parenchyma.

The aim of this study was to determine whether preservation of part of segment IV confers any clinical advantage in terms of postoperative arterial lactate concentration, length of hospital stay, rates of PHLF, renal dysfunction, and 90-day mortality compared to complete resection of segment IV up to the umbilical fissure when performing extended right hepatectomy for colorectal liver metastases (CRLM).

## 2. Materials and Methods

This study was a retrospective analysis of a prospectively maintained database of all patients undergoing right-sided liver resection for CRLM between August 2005 and March 2013. To reduce variation in the study population patients who had radio-frequency ablation (RFA) or wedge resections performed of the left lobe in addition to the main procedure were excluded from analysis. Operations were described according to the Brisbane classification [1]. Where technically possible, part of segment IV was routinely preserved when performing an extended right hepatectomy, if the retained subsegment appeared well perfused and the resection margin was not compromised. For example, subsegment IVA or B is commonly preserved when undertaking extended resection of multifocal tumours which involve only one part of segment IV. Operations were performed under low venous pressure general anaesthesia using standard techniques. Blood gas analysis was performed after closure of the abdomen and the arterial lactate concentration recorded. Serum biochemistry tests and coagulation assays were performed on patients in the first 24 postoperative hours and the tests repeated according to clinical course. PHLF was defined as an elevated prothrombin time (PT) and serum bilirubin on postoperative day five according to the ISGLS [18]. Renal dysfunction was defined as an increase in serum creatinine of ≥1.5-fold from the preoperative baseline value within the first five postoperative days, according to RIFLE criteria [19].

All patients were followed up for a minimum of 90 days and the length of hospital stay and mortality were recorded along with details of the cause of death, determined from case-sheet review, radiological and laboratory data, and death certificates. Patients who died with jaundice and/or radiological evidence of ascites and/or encephalopathy in the absence of any other clear diagnosis were determined to have died of liver failure.

The distribution of categorical variables between patient groups was compared with Fisher's exact test and continuous variables with Mann-Whitney *U* test. The significance level was set at 0.05. All analyses were performed with SPSS.

## 3. Results

A total of 117 patients who fulfilled the study criteria were identified of whom 85 had right hepatectomy (72.6%), 20 had partially extended right hepatectomy (17.1%), and 12 had fully extended hepatectomy (10.3%). Patient details and outcomes are shown in Table 1. There was no difference in the stage of the primary tumour, number of liver tumour nodules, or use of liver-directed chemotherapy between resection types, although the maximum tumour diameter was larger in patients undergoing partially extended hepatectomy or extended right hepatectomy compared to those undergoing standard right hepatectomy. Of the 69 patients who received preoperative liver-directed chemotherapy, oxaliplatin and capecitabine was the most common regime in those undergoing standard right hepatectomy (33/50), partially extended right hepatectomy (9/10), and fully extended right hepatectomy (8/9). Blood loss was higher in patients undergoing partially extended resections compared to those undergoing fully extended or standard right hepatectomies. Analysis of postoperative arterial lactate concentrations revealed a higher concentration in patients undergoing an extended hepatectomy compared to the group having a partially extended right hepatectomy or those undergoing standard right hepatectomy. This benefit in early outcome did not translate into improved late post-operative outcomes. The length of hospital stay and rates of hepatic and renal dysfunction were similar for standard compared to partially extended hepatectomy and extended right hepatectomy. There was also no significant difference in the postoperative peak in serum transaminase levels between groups. Postoperatively 11 patients developed a bile leak (9.4%) with no significant difference between groups. Both all-cause 90-day mortality and death due to liver failure were higher in patients in whom part of segment IV was preserved compared to those having a standard right hepatectomy or extended hepatectomy. There were no deaths in the group of patients having an extended right hepatectomy.

## 4. Discussion

Extended right hepatectomy is associated with significant risk due to the small size of the liver remnant. Surgeons may be presented with the option of preserving part of segment IV if a sufficient margin of resection can be obtained. The results of this study do not demonstrate any clinical benefit of this policy and suggest that an extended hepatectomy up to the umbilical fissure may produce better patient outcomes in terms of organ dysfunction and mortality.

The finding of lower postoperative arterial lactate concentration when part of segment IV is preserved in patients undergoing extended hepatectomy for CRLM is interesting and suggests that in the short term the retained subsegment of liver may provide some function, as the liver is the primary site of clearance of serum lactate [11]. This difference does not however translate into a lower incidence of liver dysfunction in the first five postoperative days compared to operations when all of segment IV is removed. The highest rates of liver failure and mortality were noted in patients who had partially extended hepatectomy compared to standard right hepatectomy and fully extended right hepatectomy. The retained part of segment IV may add up to approximately 20% of the total residual liver volume [14] and the lack of clinical benefit of preserving this subsegment may be due to the development of dysfunction and ultimately infarction due to inadequate venous drainage. The liver portion may then become a metabolic burden on the residual left lateral sector.

The strength of this study lies in the comparison of outcomes within a homogenous patient population undergoing surgery for colorectal metastases with exclusion of patients having additional procedures. The demonstration of lack of benefit of preserving part of segment IV in this patient group is useful as inevitably this procedure requires division of a larger volume of parenchyma than following a straighter resection plane close to the umbilical fissure to remove all of segment IV. This may potentially prolong the operating time and is shown to lead to greater blood loss in the current series.

The weakness of the study lies in the small sample size and the absence of formal volumetric studies. There is a potential for bias in the study in that surgeons may be more likely to attempt preservation of part of segment IV in the presence of a small left lateral sector. However, the quality of residual liver parenchyma also has an effect on postoperative outcome and both biliary obstruction [20] and hepatic steatosis [9] are major operative risks which are not measured by hepatic volumetry. We have conjectured that the lack of sufficient venous drainage contributes to dysfunction of segment IV when the middle hepatic vein is removed and that the macroscopic appearance of the retained segment may not give an accurate indication of the degree of venous drainage. This issue can be explored by the use of intraoperative ultrasound which is able to assess venous outflow by the assessment of flow dynamics within the portal vein. In the presence of outflow obstruction portal venous flow to the affected segment becomes hepatofugal [21] and this phenomenon may be visible in segment IV vessels at the umbilical fissure. This technique could potentially be used intraoperatively to determine the vascular integrity of hepatic parenchyma close to resection margins.

Recent trends in liver surgery have raised the awareness of preserving hepatic parenchyma both to improve short-term outcomes and to allow for repeat resections. However, the data in this study demonstrate that preservation of hepatic volume may not lead to improved outcome when the function of marginal areas of preserved liver is compromised. This has implications for other areas of liver surgery where nonanatomic procedures may be undertaken in an attempt to retain the maximum volume of liver parenchyma.

## Figures and Tables

**Table 1:** Patient characteristics, operative details, and outcomes for 117 patients undergoing right-sided hepatectomy for colorectal liver metastases.

*N* = 117	Operation						*P* value	
	Right hepatectomy (*n* = 85)		Partially extended right hepatectomy (*n* = 20)		Extended right hepatectomy (*n* = 12)			
	Count (%)	Median (range)	Count (%)	Median (range)	Count (%)	Median (range)	Partially extended versus right	Extended right versus right
Age		65 (33–82)		66 (40–88)		61 (54–77)	0.899	0.354
Gender								
Female	35 (41.2)		7 (35.0)		5 (41.7)		0.800	1.000
Male	50 (58.8)		13 (65.0)		7 (58.3)			
T stage of primary								
0	1 (1.2)		1 (5.0)		0		0.326	1.000
1	2 (2.4)		0		0		1.000	1.000
2	4 (4.7)		2 (10.0)		2 (16.7)		0.290	0.144
3	48 (56.5)		12 (60.0)		6 (50.0)		0.600	1.000
4	28 (32.9)		3 (15.0)		3 (25.0)		0.258	1.000
N/A*	2 (2.4)		2 (10.0)		1 (8.3)		—	—
N stage of primary								
0	39 (45.9)		7 (35.0)		3 (25.0)		0.608	0.335
1	21 (24.7)		7 (35.0)		6 (50.0)		0.257	0.072
2	23 (27.1)		4 (20.0)		2 (16.7)		0.774	0.721
N/A*	2 (2.4)		2 (10.0)		1 (8.3)		—	—
Preoperative Chemotherapy								
Yes	50 (58.8)		10 (50.0)		9 (75.0)		0.616	0.356
No	35 (41.2)		10 (50.0)		3 (25.0)			
Number of chemotherapy cycles		4 (2–11)		4 (1–4)		5 (1–6)	0.115	0.854
Synchronous bowel resection	1 (1.2)		0		1 (8.3)		1.000	0.233
Number of tumours		1.5 (1–9)		2 (1–7)		2.5 (1–8)	0.100	0.118
Maximum diameter (mm)		35 (5–120)		50 (11–155)		52.5 (17–150)	0.026^†^	0.004^†^
Pringle manoeuvre used	0		1 (5.0)		0		0.191	1.000
Blood loss								
<500 mL	43 (50.6)		5 (25.0)		4 (33.3)		0.046^†^	0.357
>500 mL	41 (48.2)		15 (75.0)		8 (66.7)			
Not recorded	1 (1.2)		0		0			
Postoperative lactate (mmol/L)		3.3 (1.2–15.0)		3.35 (1.7–11.6)		4.35 (2.4–5.2)	0.592	0.006^†^
Bile leak	7 (8.2)		2 (10.0)		2 (16.7)		0.680	0.308
Renal dysfunction (POD 0–5)								
Yes	10 (11.8)		3 (15.0)		0		0.710	0.355
No	75 (88.2)		17 (85.0)		12 (100)			
Peak postoperative transaminase (iu/L)		376 (104–1557)		435 (92–2331)		309 (81–921)	0.356	0.289
PHLF on POD 5								
Yes	13 (15.3)		4 (20.0)		1 (8.3)		0.508	1.000
No	72 (84.7)		15 (75.0)		11 (91.7)			
N/A**			1 (5.0)					
Length of stay (days)		8 (4–30)		8 (6–27)		8 (5–15)	0.613	0.629
90-day mortality								
Yes	3 (3.5)		4 (20.0)		0		0.024^†^	1.000
No	82 (96.5)		16 (80.0)		12 (100)			
Death from liver failure								
Yes	2 (2.4)		3 (15.0)		0		0.047^†^	1.000
No	83 (97.6)		17 (85.0)		12 (100)			

*Primary pathology not available for 5 patients. **PHLF not applicable if patient died before POD 5. ^†^Significant at the level of *P* < 0.05.
